# Supplementary material for: Iterative improvement in the automatic modular design of robot swarms
Source: PeerJ Comput Sci. 2020 Dec 7;6:e322. doi: 10.7717/peerj-cs.322 (PMC7924708; doi:10.7717/peerj-cs.322)
Supplement: Supplemental Information 3 [file peerj-cs-06-322-s003.zip › argos3/doc/api/standalone/a00431_source.html]

ARGoS: plugins/robots/eye-bot/simulator/pointmass3d\_eyebot\_model.h Source File


- Main Page
- Related Pages
- Namespaces
- Classes
- Files

- File List
- File Members

# plugins/robots/eye-bot/simulator/pointmass3d\_eyebot\_model.h

Go to the documentation of this file.

```
00001 
00007 #ifndef POINTMASS3D_EYEBOT_H
00008 #define POINTMASS3D_EYEBOT_H
00009 
00010 namespace argos {
00011    class CPointMass3DEngine;
00012    class CPointMass3DQuadRotorModel;
00013    class CPointMass3DEyeBotModel;
00014    class CQuadRotorEntity;
00015 }
00016 
00017 #include <argos3/plugins/simulator/physics_engines/pointmass3d/pointmass3d_quadrotor_model.h>
00018 #include <argos3/plugins/robots/eye-bot/simulator/eyebot_entity.h>
00019 
00020 namespace argos {
00021 
00022    class CPointMass3DEyeBotModel : public CPointMass3DQuadRotorModel {
00023 
00024    public:
00025       
00026       CPointMass3DEyeBotModel(CPointMass3DEngine& c_engine,
00027                               CEyeBotEntity& c_eyebot);
00028       virtual ~CPointMass3DEyeBotModel() {}
00029 
00030       void UpdatePerspectiveCameraAnchor(SAnchor& s_anchor);
00031       
00032    };
00033 
00034 }
00035 
00036 #endif
```

---

Generated on 10 Jul 2018 for ARGoS by 
 1.6.1
